# Impact of the Herbal Medicine *Sophora flavescens* on the Oral Pharmacokinetics of Indinavir in Rats: The Involvement of CYP3A and P-Glycoprotein

**DOI:** 10.1371/journal.pone.0031312

**Published:** 2012-02-16

**Authors:** Jia-Ming Yang, Siu-Po Ip, Yanfang Xian, Ming Zhao, Zhi-Xiu Lin, John Hok Keung Yeung, Raphael Chiu Yeung Chan, Shui-Shan Lee, Chun-Tao Che

**Affiliations:** 1 School of Chinese Medicine, The Chinese University of Hong Kong, Shatin, Hong Kong; 2 School of Biomedical Science, The Chinese University of Hong Kong, Shatin, Hong Kong; 3 Department of Microbiology, The Chinese University of Hong Kong, Shatin, Hong Kong; 4 Stanley Ho Centre for Emerging Infectious Diseases, The Chinese University of Hong Kong, Shatin, Hong Kong; 5 Department of Medicinal Chemistry and Pharmacognosy, University of Illinois at Chicago, Chicago, Illinois, United States of America; University of Rochester, United States of America

## Abstract

*Sophora flavescens* is a Chinese medicinal herb used for the treatment of gastrointestinal hemorrhage, skin diseases, pyretic stranguria and viral hepatitis. In this study the herb-drug interactions between *S. flavescens* and indinavir, a protease inhibitor for HIV treatment, were evaluated in rats. Concomitant oral administration of *Sophora* extract (0.158 g/kg or 0.63 g/kg, p.o.) and indinavir (40 mg/kg, p.o.) in rats twice a day for 7 days resulted in a dose-dependent decrease of plasma indinavir concentrations, with 55%–83% decrease in AUC_0-∞_ and 38%–78% reduction in C_max_. The CL (Clearance)/F (fraction of dose available in the systemic circulation) increased up to 7.4-fold in *Sophora*-treated rats. Oxymatrine treatment (45 mg/kg, p.o.) also decreased indinavir concentrations, while the ethyl acetate fraction of *Sophora* extract had no effect. Urinary indinavir (24-h) was reduced, while the fraction of indinavir in faeces was increased after *Sophora* treatment. Compared to the controls, multiple dosing of *Sophora* extract elevated both mRNA and protein levels of P-gp in the small intestine and liver. In addition, *Sophora* treatment increased intestinal and hepatic mRNA expression of CYP3A1, but had less effect on CYP3A2 expression. Although protein levels of CYP3A1 and CYP3A2 were not altered by *Sophora* treatment, hepatic CYP3A activity increased in the *Sophora*-treated rats. All available data demonstrated that *Sophora flavescens* reduced plasma indinavir concentration after multiple concomitant doses, possibly through hepatic CYP3A activity and induction of intestinal and hepatic P-gp. The animal study would be useful for predicting potential interactions between natural products and oral pharmaceutics and understanding the mechanisms prior to human studies. Results in the current study suggest that patients using indinavir might be cautioned in the use of *S. flavescens* extract or *Sophora*-derived products.

## Introduction

Dried root of *Sophora flavescens* Ait. (Fabaceae), known in Chinese as Ku-Shen, is widely used in traditional Chinese Medicine as a “cooling” drug for the treatment of gastrointestinal hemorrhage, skin diseases, and pyretic stranguria. The herb is an antipyretic, anthelminitic and diuretic agent [1]. Phytochemical studies have revealed the presence of quinolizidine alkaloids and prenylated flavonoids [2], [3], both of which show a wide spectrum of pharmacological activities, such as antitumor, cardioprotective, and neuroprotective effects [4]–[6]. The alkaloids, such as matrine and oxymatrine, are frequently used as the chemical markers for the quality control of this medicinal herb. Pharmaceutical products derived from these alkaloids, such as the Marine Capsule [Jiangsu Chia-Tai Tianqing Pharmacy Co., China] (which contains mainly oxymatrine and will be referred to as “oxymatrine capsule” thereafter), are available for the treatment of viral hepatitis in China.

The widespread use of *S. flavescens* as complementary or alternative medicine has led to increasing concerns on potential herb-drug interactions through effects on enzyme pathways. It is known that Cytochrome P450 (CYP)-dependent monooxygenase system plays a primary role in the elimination of a variety of xenobiotics and is often a major component of drug-drug and herb-drug interactions. *Sophora* treatment has been shown to cause CYP1A induction and male-specific induction of CYP2A and CYP3A in mice, and it has been suggested that oxymatrine contributed at least partly to the CYP induction by *S. flavescens* [7]. A further study demonstrated that *S. flavescens* reduced theophylline plasma levels, which may be related to the inductions of CYP1A2, CYP2B1/2, CYP2C11, and CYP3A [8]. Matrine and oxymatrine, the two major pharmacologically active ingredients, have been shown to cause CYP2B induction but not CYP3A, possibly mediated by the activation of constitute androstane receptor (CAR) [9]. More recently, a mechanistic study using cell-based gene reporter assays demonstrated that *S. flavescens* induced CYP3A expression through the activation of pregnane X receptor (PXR), and *N*-methylcytisine, an alkaloid constituent of *S. flavescens*, was identified to be a potent PXR activator [10]. Taken together, these findings suggest that there may be potential herb-drug interaction when *S. flavescens* is used, especially in conditions involving the prescription of multiple medicinal compounds, for example human immunodeficiency virus (HIV) infection and acquired immune deficiency syndrome (AIDS).

Indinavir is a potent and specific protease inhibitor (PI) which has long been used as a component of the highly active antiretroviral therapy (HAART) for the treatment of HIV/AIDS. Although new drugs have recently been developed, indinavir remains a popular choice in many developing countries. The metabolism of indinavir has been extensively studied in human, rats, dogs, and monkeys [11]–[14]. It is metabolized predominantly via CYP3A and is also a substrate of P-glycoprotein (P-gp), an efflux transporter that can limit the oral absorption of indinavir. Consequently, co-administration of foods, drugs or dietary supplements with influences on CYP3A and/or P-gp may affect the pharmacokinetics of indinavir, causing undesirable toxicity and/or diminishment of drug efficacy. Piscitelli *et al.* [15] reported that the plasma levels of indinavir were reduced in healthy volunteers after co-administration of St. John's wort. A recent study in rats confirmed that St. John's wort decreased plasma indinavir concentrations, and the interaction was attributed to the induction of indinavir metabolism in liver and intestine [16]. The therapeutic range of PIs is relatively narrow, and the antiretroviral activity correlates closely to their plasma concentrations [17]. Low plasma concentrations of PIs can cause viral resistance and treatment failure [18], and conversely, elevated plasma concentrations may enhance drug toxicity [19].

Given the shared transmission routes, HIV patients are vulnerable to multiple viral co-infections such as that of hepatitis B virus (HBV) and hepatitis C virus (HCV) [20], [21]. Matrine and oxymatrine, two major components of *S. flavescens*, are widely used in the treatment of chronic viral hepatitis in China [22], [23]. *Sophora*-derived products such as the Marine Capsule are often taken by HBV- or HCV-coinfected HIV patients while they are receiving indinavir. Against these backgrounds, the current study aims to establish an animal model to evaluate potential herb-drug interaction between *Sophora flavescens* and indinavir, and to explore the possible underlining mechanisms. To mimic the situations in clinical practice, rats were orally administered with indinavir and *S. flavescens* extract, or its ingredients, concomitantly for seven consecutive days. Plasma concentration profile and urinary and faecal excretion of indinavir were then determined. The possible involvement of P-gp and CYP3A in this interaction was examined by measuring the intestinal and hepatic mRNA/protein levels and enzyme activity.

## Materials and Methods

### Preparation of *Sophora* Extract

Dried roots of *Sophora flavescens* (500 g, Batch Number: 001110), provided by Zhixin Pharmaceutical Company (Guangdong, China), were authenticated by Miss Yuying Zong using pharmacopoeial procedures (Chinese Pharmacopoeia, 2010) [1]. Voucher samples (HHSF-SF-001) have been deposited at the School of Chinese Medicine, the Chinese University of Hong Kong. The pulverized materials were soaked in 70% ethanol (4 L) for 30 min at room temperature, followed by boiling three times (1 h each) in 4 L, 3 L and 3 L of 70% ethanol, successively. The combined extract was filtered and concentrated by rotary evaporation under reduced pressure. Finally, the concentrate was freeze-dried to obtain a powder (31.6% yield from the dried plant material), which contained approximately 11.1% oxymatrine (by HPLC-UV determination at 220 nm). Part of the *Sophora* extract powder (c.a. 50 g) was suspended in hot water (200 mL) and partitioned with equal volume of ethyl acetate (EA) for three times. The organic layers were combined, evaporated under reduced pressure, and freeze-dried. The yield of EA fraction was 13% from *Sophora* total extract and the oxymatrine content in the EA fraction was less than 0.02%. The dried powder was stored in a desiccator at room temperature until use. Marine Capsule (containing >95% oxymatrine) was purchased from Chia-Tai Tianqing Pharmacy Co. Ltd. (Jiangsu, China).

### Animals

Male Wistar rats (230–260 g) were bred and housed by the Laboratory Animal Services Centre of the Chinese University of Hong Kong. All experiments were performed with approval from the Animal Research Ethics Committee of the Chinese University of Hong Kong. A License to Conduct Experiments was obtained from the Department of Health, the Hong Kong Special Administrative Region Government [Ref No: (09-515) in DH/HA&P/8/2/1 Pt.10]. The animals were kept in a temperature-controlled room (23±2°C) with a 12-h light-dark cycle, with free access to food and water.

### Pharmacokinetic Studies of Indinavir

Thirty rats were randomly designated as vehicle control, low-dose and high-dose *Sophora* treatment groups (ten rats in each group). A separate experiment was performed using eighteen rats designated as vehicle control, oxymatrine capsule and *Sophora* EA fraction groups (six rats in each group). The *Sophora* extract and EA fraction powders were homogenized in 1.5% Tween 80 with a mortar and pestle. Indinavir from CRIXIVAN® capsule (Merck & CO. Inc., Whitehouse Station, NJ, USA) was dissolved in 50 mM citric acid, and the oxymatrine capsule contents were dissolved in distilled water. Rats were gavaged with 1.5% Tween 80 (vehicle), *Sophora* extract (0.158 g/kg or 0.63 g/kg), oxymatrine capsule (45 mg/kg of oxymatrine equivalent), or *Sophora* EA fraction (82 mg/kg), respectively. To avoid possible physiochemical influence on indinavir absorption, indinavir (40 mg/kg) was given to all animals by gavage an hour later. The treatments were given twice a day (9 a.m. and 7 p.m.) for 7 consecutive days. The dosages were designed on the basis of clinical doses in human. The daily dose of *S. flavescens* recommended by the Chinese Pharmacopoeia is 4.5–9 g of raw herb per day [1]. The “Kushen Pian” (literally meaning “*Sophora* tablet”), a *Sophora*-derived formulated product for the treatment of eczema, is used at a dose equivalent to 12–18 g of raw herb per day [24]. Taking the low and high ends of these dose ranges, and using a conversion based on the body-surface-area ratio between rats and human, an animal dose range of 0.45–1.8 g/kg/day (raw herb equivalent) was estimated for experimental rats. Subsequently, two dose levels, 0.316 and 1.26 g/kg/day, were selected for use in the present study to represent the low- and high-dose levels.

To study the excretion of indinavir, the rats were placed in individual metabolic cages after the second daily treatment (8 p.m.) on the sixth dosing day, and urine and faeces samples were collected until 8 p.m. on day 7. On the experimental day (day 8), animals were treated with the last dose of vehicle or *Sophora* preparations, and thereafter indinavir. Under anesthetization with isoflurane, blood samples (0.3 mL) were collected before indinavir dosing and at 0.25, 0.5, 1, 1.5, 2, 3, 4 and 5 h after dosing by orbital bleeding via heparinized capillary tubes. Plasma was obtained by centrifugation at 7,000× g for 15 min at 4°C and frozen at −80°C prior to analysis. After the last sampling at 5 h, rats were sacrificed by cervical dislocation. Organ tissues, including liver and intestine, were isolated, rinsed with saline, blotted dried, snap-frozen in liquid nitrogen, and stored at −80°C until use.

### Measurement of Hepatic and Intestinal mRNA

The amounts of mRNA encoding CYP3A1, CYP3A2 and P-gp (mdr1a and mdr1b) in the intestine and liver were quantified by real-time PCR. The tissues (<100 mg) were homogenized in 1 mL Trizol reagents (Invitrogen, Carlsbad, CA, USA). Total RNA was extracted using chloroform and isopropanol. The RNA was quantified by the standard OD_260_ method. The OD_260_/OD_280_ ratio for each RNA sample ranged from 1.8 to 2.2. Subsequently, RNA was converted to cDNA using high-capacity cDNA reverse transcription kit (Applied Biosystems, Carlsbad, CA, USA) according to the manufacturer's instructions. Taqman assays were performed for the quantification of mRNA in an ABI step-one real time PCR system (Applied Biosystems, , Carlsbad, CA, USA). Specific primers for CYP3A1, CYP3A2, mdr1a, mdr1b and the housekeeping gene *β*-actin were purchased from Applied Biosystems (product codes: Rn01412959_g1, Rn00756461_m1, Rn01639253_m1 and Rn00561753_m1, respectively). The relative mRNA levels were calculated by the 2^−ΔΔCT^ method [25].

### Measurement of Intestinal and Hepatic CYP3A and P-gp Protein Expression

Intestinal brush border membrane (BBM), intestinal mucosa homogenate and liver microsomes were used for protein analysis. The frozen small intestine (ileum and jejunum) was cut open on a glass plate. Intestinal mucosa was collected by scraping and homogenized with a glass tube homogenizer (striking with a loose fitting pestle) in lysis buffer (50 mM Tris-HCl *p*H 7.4, 150 mM NaCl, 1% v/v NP-40, 0.5% sodium deoxycholate, and 1% 100× protease inhibitor mixture (GE Healthcare, Waukesha, WI, USA). The homogenate was divided to two portions. One portion was allowed to settle on ice for 30 min after homogenization, and then centrifuged at 10,000× g for 20 min. The resulting supernatant was collected for the analysis of intestinal CYP3A. The other portion was used for the preparation of intestinal BBM according to a modified calcium precipitation method [26]. CaCl_2_ was added to a final concentration of 10 mM to the homogenate. After settling on ice for 30 min, the homogenate was centrifuged at 3,000× g for 15 min, and the resulting supernatant was further centrifuged at 24,000× g for 40 min. The pellet was resuspended in lysis buffer for the analysis of intestinal P-gp.

Liver microsomes were prepared by a differential centrifugation method. Briefly, 5 g of frozen liver was homogenized in 0.1 M potassium phosphate buffer (*p*H 7.4; 10 mL), containing 3.3 mM MgCl_2_, with a high-speed homogenizer. The homogenates were centrifuged at 9,000 g for 30 min, and the resulting supernatants were further centrifuged at 100,000× g for 60 min. The pellet was rinsed with potassium phosphate buffer, spinned down at 100,000× g and resuspended in buffer (0.1 M potassium phosphate, *p*H 7.4, 3.3 mM MgCl_2_ and 250 mM sucrose) for the analysis of hepatic CYP3A and P-gp.

Protein contents were quantified by the Bio-Rad (Hercules, CA, USA) protein assay kit. Samples of intestinal BBM (80 µg for P-gp), intestinal mucosa supernatant (20 µg for CYP3A), or liver microsome (10 µg for CYP3A and 80 µg for P-gp) were resolved in 8% or 12% SDS-polyacrylamide gel (Mini-PROTEIN 3 System, Bio-Rad) and transferred to polyvinylidene difluoride membranes, which were blocked for 1.5 h in a buffer containing 5% nonfat milk, 150 mM NaCl, 15 mM Tris (pH 7.6), and 1 mM EDTA. The membranes were then probed with anti-P-gp monoclonal antibody C219 (1∶1000, EMD Chemicals, Gibbstown, NJ, USA), specific anti-CYP3A1 or anti-CYP3A2 polyclonal antibody (1∶4000, Millipore, Billerica, MA, USA), or anti-ERp29 polyclonal antibody as control (1∶5000, Thermo Scientific, Waltham, MA, USA) in Tris-buffered saline containing 0.05% Tween 20 at 4°C overnight. After washing three times with Tris-buffered saline (10 min each), the membranes were incubated with horseradish peroxidase-conjugated secondary antibody (1∶5000, Cell Signaling Technology, Danvers, MA, USA) for 1 h at room temperature. After the blots were washed three times with Tris-buffered saline, the immunoreactive bands were detected with the enhancing chemiluminescence plus detection system (Amersham Pharmacia Biotech Inc., Piscataway, NJ, USA), and visualized on X-ray films. Band intensity was then scanned and quantified using the Gel-Pro Analyzer 4.0 software (Media Cybernetics, Bethesda, MD, USA). The P-gp, CYP3A1 and CYP3A2 intensities were normalized to that of ERp29.

### CYP3A Activity Assay

The hepatic CYP3A activity was measured by a luminescent assay (P450-Glo) according to the manufacturer's instructions (Promega Corporation, Madison, WI, USA). Briefly, rat liver microsomes were prepared using differential centrifugation described above. The CYP3A catalytic reaction was performed in 200 mM potassium phosphate buffer solution (*p*H 7.4). A reaction mixture, containing microsomal protein (0.8 mg/mL) and Luciferin-BE substrate (50 µM), was pre-incubated at room temperature for 10 min. The reaction was initiated by adding NADPH-regenerating system (β-NADP^+^, glucose-6-phosphate and glucose-6-phosphate dehydrogenase). After 20-min incubation, Luciferin detection reagent was added and allowed for 20-min incubation. Luminescence, which was in proportion to CYP3A activity, was measured by a luminometer. The relative CYP3A activity was expressed as the fold over control.

### Analytical Methods

Concentrations of indinavir in the biological specimens were determined by HPLC-MS according to previous reports [27], [28] with minor modifications. Midazolam was used as the internal standard. Plasma and urine samples (100 µL) were alkalized by 50 mM NaOH solution after addition of midazolam (20 µL, 50 µg/mL), and then extracted with ethyl acetate (1 mL) for 15 min. The organic layers were transferred to glass tubes, and dried under nitrogen at 45°C. The residues were reconstituted with 200 µL of methanol-water (v/v, 1∶1) solution. The faeces were homogenized in about 4-fold volume of distilled water. The homogenates were extracted with equal volume of acetonitrile. After centrifugation, 1 mL of the supernatant was transferred to a glass tube, and was subsequently subjected to addition of midazolam, alkalization, extraction with ethyl acetate, drying and reconstitution. All reconstituted samples (60 µL) were separated on a Prevail C_18_ column (250 mm×4.6 mm I.D., 5 µm, Alltech) by an Agilent 1100 HPLC system (Agilent Technologies, Santa Clara, CA, USA). The flow rate was 0.8 mL/min. The mobile phase consisted of 0.1% trifluoroacetic acid water (A) and acetonitrile (B) programmed to a gradient elution of 20–37% B at 0–13 min. The effluent was introduced to APCI-MS (Agilent 1100 series LC/MSD Trap SL system, Santa Clara, CA, USA) with positive detection mode. The scan range was set from *m/z* 150–900 and the target mass was *m/z* 470. MS conditions were optimized as follows: nebulizer (N_2_), 50 psi; dry gas (N_2_), 5 L/min; drying gas temperature, 325°C; compound stability, 100%; trap drive level, 100%. Extract ion chromatograms at *m/z* 614 (indinavir) and *m/z* 326 (midazolam) were used for quantification (Figure S1). Quantification of indinavir was performed on six levels of standards in blank plasma or faeces homogenate. The calibration curves showed good linear correlation (*r^2^*≥0.99) between peak area ratio (indinavir over midazolam) and concentrations. Intra- and inter-day precision of the method was within the acceptable limits with R.S.D.<10%. Samples were stable for 72 h at room temperature (R.S.D.<5%). The recovery of the analyte ranged from 80% to 90%.

### Pharmacokinetic and Statistical Analysis

Pharmacokinetic parameters of indinavir were determined by a noncompartmental pharmacokinetic model using the PK Solutions 2.0.2 software (Summit Research Services, Ashland, OH, USA). The area under the plasma concentration-time curves from zero to infinity (AUC_0-∞_) was calculated by the trapezoidal rules. Clearance (CL) was calculated by the equation (F×Dose)/AUC_0-∞_, where F is the fraction of dose available in the systemic circulation. Peak plasma concentration (C_max_) and the time when it occurred (*T*_max_) were derived directly from the curves. Statistical differences were determined by unpaired Student's *t* test or one-way analysis of variance (one-way ANOVA) followed by Dunnett's post test. All tests were two-tailed and the significance was set at *P*<0.05.

## Results

### Effect of *Sophora flavescens* on Plasma Indinavir Concentrations

**Figure 1:**
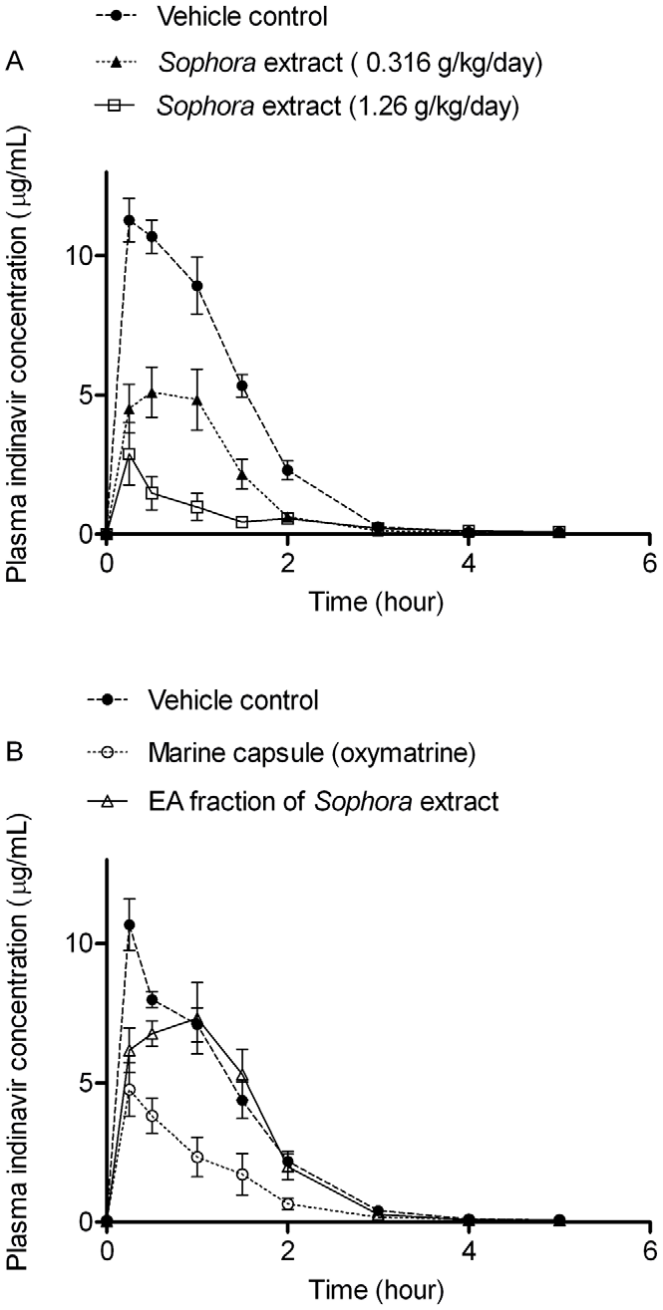
Time course of the plasma concentration of indinavir. Animals received oral administration of indinavir (40 mg/kg) together with (A) 1.5% Tween 80 (vehicle), 70% ethanol extract of *Sophora flavescens* (0.158 g/kg and 0.63 g/kg), (B) Oxymatrine capsule (45 mg/kg of oxymatrine equivalent), or ethyl acetate (EA) fraction of *Sophora* extract (0.082 g/kg), twice a day for 7 days, respectively. On the experimental day (day 8), animals were pretreated with corresponding vehicle or tested drugs at 1 h prior to the last dose of indinavir (40 mg/kg) given to all rats for pharmacokinetic study. Values are expressed as mean ± S.E.M. for each data point (n = 6–9).

The effect of *Sophora* extract on the multiple-dose pharmacokinetics of indinavir was assessed by measuring the plasma concentrations. The plasma concentration profiles of indinavir on the experiment day (day 8), with or without *Sophora* co-treatment, are shown in Figure 1(A). At time zero, plasma indinavir concentration was detectable, but was much lower than the limit of quantification (50 ng/mL). The pharmacokinetic parameters of the control and treatment groups are shown in Table 1. Maximum plasma concentration (C_max_) in vehicle-treated rats was 11.54 µg/mL, while that in the *Sophora*-treated rats decreased dose-dependently by 38% (*P*<0.01) and 78% (*P*<0.001) in the low-dose (0.316 g/kg per day) and high-dose groups (1.26 g/kg per day), respectively. The AUC_0-∞_ of indinavir in *Sophora*-treated rats decreased as well, showing 55% (*P*<0.001) and 83% (*P*<0.001) reduction in the respective treatment group. Meanwhile, *Sophora*-treated rats displayed significantly higher CL/F value than the control, with dose-dependent increases by 2.4-fold (*P*<0.001) and 7.4-fold (*P*<0.001) in the low- and high-dose groups, respectively. On the other hand, the *T*_max_ value was not significantly altered after *Sophora* treatment.

**Table 1:** Pharmacokinetic parameters of indinavir (40 mg/kg orally) after multiple dosage.

	Vehicle control	*Sophora* extract		Oxymatrine (90 mg/kg/day)	EA fraction (0.164 g/kg/day)
		0.316 g/kg/day	1.26 g/kg/day		
AUC_0-∞_ (µg·h/mL)	16.07±0.99	7.23±0.87***	2.78±0.61***	5.29±1.11***	12.27±1.31
C_max_ (µg/mL)	11.54±0.67	7.17±0.69**	3.09±1.08***	4.97±0.81***	8.41±0.86
*T*_max_ (h)	0.50±0.10	0.62±0.12	0.50±0.19	0.42±0.12	0.67±0.15
CL/F (mL/h/kg)	2570±183	6047±693***	18958±3513***	9569±2302*	3459±421

The rats received oral administration of indinavir (40 mg/kg) together with 1.5% Tween 80 (vehicle), *Sophora* extract (0.158 g/kg or 0.63 g/kg), Oxymatrine capsule (45 mg/kg of oxymatrine equivalent), or ethyl acetate (EA) fraction of *Sophora* extract (0.082 g/kg), twice a day for 7 days, respectively. On the experimental day (day 8), animals were pretreated with corresponding vehicle or tested drugs at 1 h prior to the last dose of indinavir given to all rats for pharmacokinetic study. Values are expressed as mean ± S.E.M. (n = 6–9). Significance is indicated as

*P<0.05,

**P<0.01,

***P<0.001, compared to vehicle control.

In a separate experiment, the effects of oxymatrine capsule (containing >95% oxymatrine) and *Sophora* EA fraction on indinavir pharmacokinetics were studied. As shown in Figure 1(B), plasma indinavir concentrations were reduced when co-administered with the oxymatrine capsule (equivalent to 90 mg/kg/day of oxymatrine). Compared to the control, the AUC_0-∞_ and C_max_ values in the oxymatrine-treated rats were decreased by 61% (*P*<0.001) and 54% (*P*<0.001), respectively (Table 1). Oxymatrine capsule treatment also increased the CL/F value by 2.2-fold (*P* = 0.017). In contrast, treatment of rats with *Sophora* EA fraction, at a dose (0.164 g/kg/day) equivalent to 1.26 g/kg/day of *Sophora* total extract, had no effect on indinavir pharmacokinetics, not affecting the AUC_0-∞_, C_max_, *T*_max_ or CL/F values. An HPLC-MS analysis revealed that prenylated flavonoids were present in the *Sophora* EA fraction, while the other fractions contained quinolizidine alkaloids including matrine and oxymatrine (Figure S2).

### Effect of *Sophora* Extract on Indinavir Excretion

**Figure 2:**
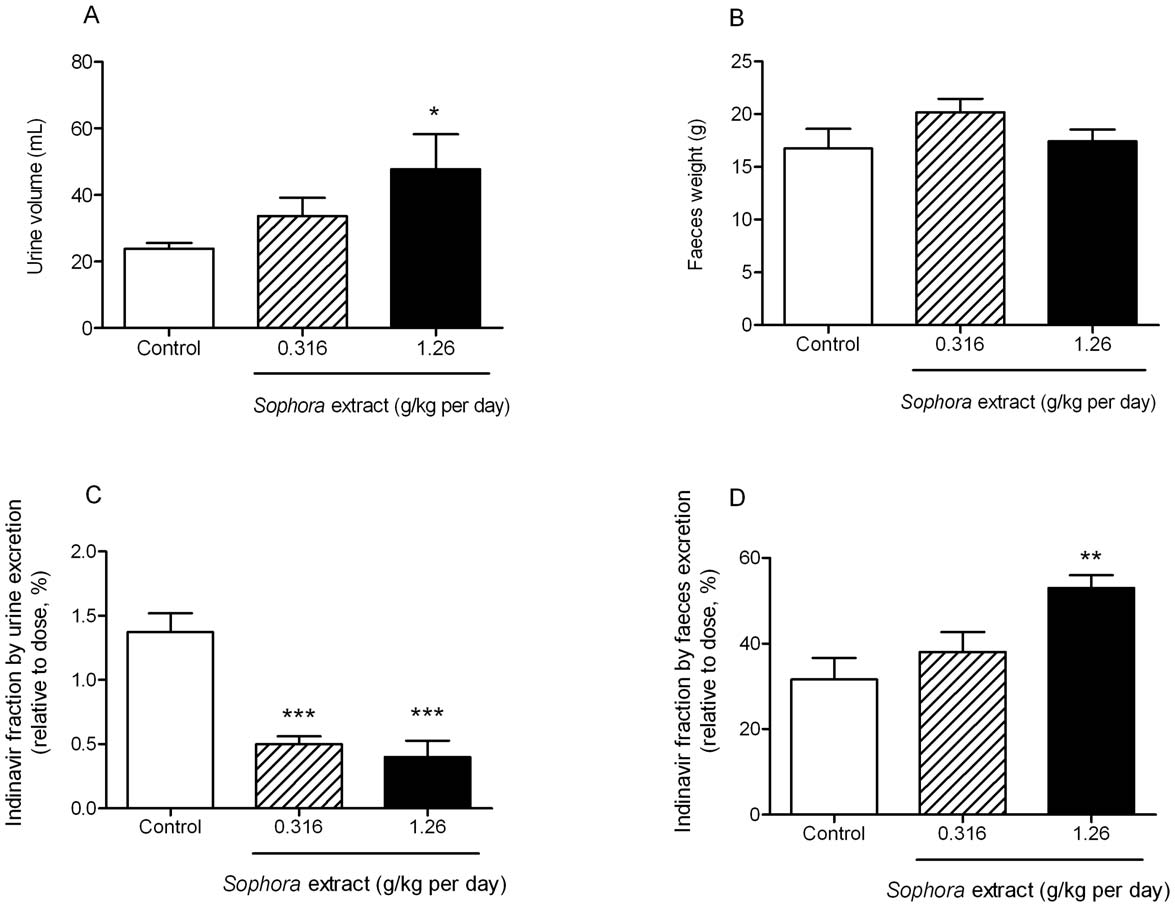
Effect of *Sophora* extract on the output of urine and faeces, and on indinavir excreted via urine and faeces. (A) Urine output, (B) Faeces output, (C) Indinavir excreted in urine, (D) Indinair excreted in faeces. The urine and faeces were collected from 8 p.m. on day 6 (after the second daily treatment) to 8 p.m. on day 7 (before the second daily treatment). Values are expressed as mean ± S.E.M. (n = 8–9).

Urine and faeces were collected from day 6 to day 7 (for 24 h) to examine the effect of *Sophora* extract on indinavir excretion. Figure 2(A) shows that *Sophora* treatment enhanced urine output dose-dependently with up to 2-fold increase in high-dose group as compared to the control, which reflects the diuretic effect of *S. flavescens*. However, the fractions of indinavir found in the urine relative to the doses given to animals was, respectively, reduced by 63% and 71% in low-dose and high-dose groups (*P*<0.001, Figure 2(C)). There was no significant alteration in faecal outputs after *Sophora* treatment (Figure 2(B)), whereas the fraction of indinavir found in the faeces (relative to dose) from rats treated with high *Sophora* dose was 1.7-fold higher than that in the control rats (*P* = 0.021, Figure 2(D)).

### Effect of *Sophora* Extract on CYP3A Expression in the Intestine and Liver

**Figure 3:**
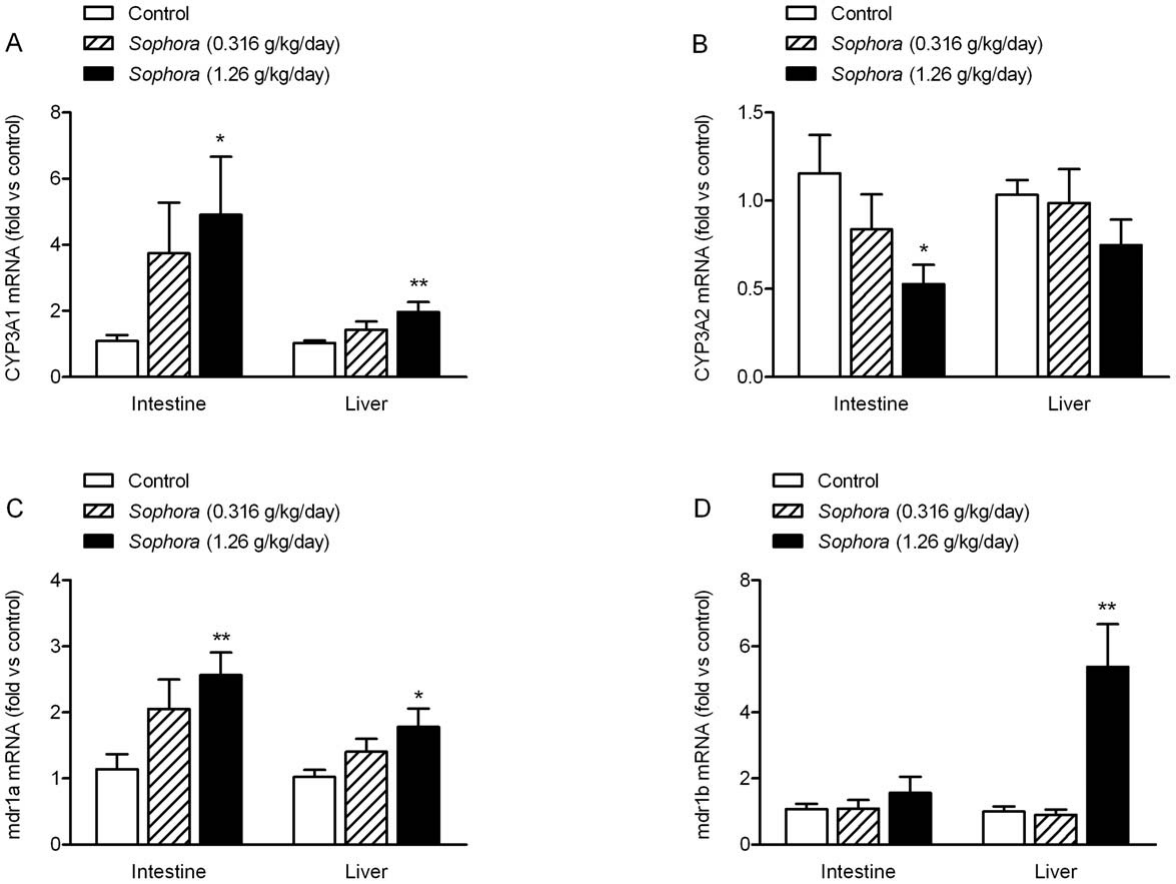
Effect of *Sophora* extract on the intestinal and hepatic mRNA levels encoding CYP3A1, CYP3A2, mdr1a and mdr1b. (A) CYP3A1, (B) CYP3A2, (C) mdr1a, (D) mdr1b. The mRNA contents were measured by real-time PCR and calculated as comparative levels over control using the 2^−ΔΔCT^ method (mean ± S.E.M., n = 7–9). Statistical significance is indicated as * *P*<0.05, ** *P*<0.01, compared to control.

To determine the effect of 7-day co-administration of *Sophora* extract on the expression of intestinal and hepatic CYP3A, mRNA and protein levels were measured by real-time PCR and Western blotting analysis, respectively. The mRNA levels of β-actin indicated by C_T_ values did not change significantly between control group and treatment groups, thus it was used as the endogenous control for calculating relative mRNA expression. Specific primers for CYP3A1 or CYP3A2 were used for the initiation of polymerase chain reaction. In the small intestine, mRNA level of CYP3A1 was found to be higher than that of CYP3A2, as evidenced by the lower C_T_ value for CYP3A1 (data not shown). Figure 3(A) shows a dose-dependent increase in the intestinal mRNA levels of CYP3A1 in *Sophora*-treated rats, with 2.4-fold (*P* = 0.053) and 3.4-fold (*P* = 0.027) enhancement in the low- and high-dose groups, respectively. In contrast, *Sophora* treatment appeared to decrease the intestinal mRNA level of CYP3A2; and dosing with 1.26 g/kg/day *Sophora* extract showed a 54% reduction (*P* = 0.033, Figure 3(B)). In liver, mRNA levels of CYP3A1 and CYP3A2 were comparable (data not shown). Compared to the control, treatment with 1.26 g/kg/day *Sophora* extract significantly increased the hepatic mRNA levels of CYP3A1 by 96% (*P* = 0.006, Figure 3(A)). On the other hand, the hepatic mRNA level of CYP3A2 was not significantly influenced by *Sophora* treatment, although there was a slight decline in the high-dose group (*P* = 0.101).

**Figure 4:**
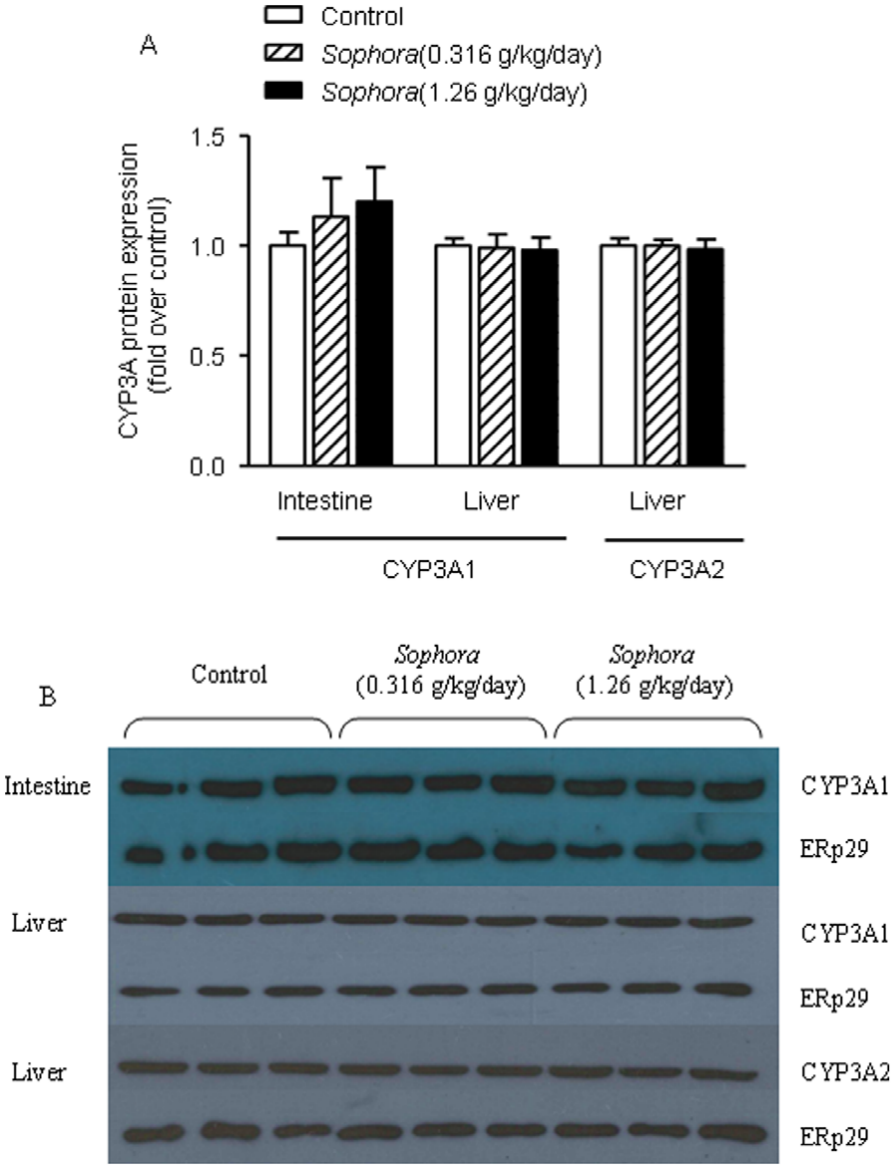
Effect of *Sophora* extract on the expression of intestinal and hepatic CYP3A1 and CYP3A2. (A) Statistical data analysis of Western blotting results, (B) image of Western blotting results. CYP3A1 and CYP3A2 levels were measured respectively using the specific anti-rat CYP3A1 and CYP3A2 antibodies. ERp29 was used as control for the normalization of CYP3A density. Bars represent mean ± S.E.M. of fold relative to the values in the control group (n = 7–8). No significant difference in the expression of either intestinal or hepatic CYP3A was observed.

The CYP3A1 and CYP3A2 antibodies specifically recognized a protein band of 58 kDa and 53 kDa, respectively. As shown in Figure 4(B), both CYP3A1 and CYP3A2 could be detected in the liver samples, while in the intestinal mucosa, only CYP3A1 could be detected. In contrast to the mRNA results, treatment of *Sophora* extract did not alter the protein levels of CYP3A1 or CYP3A2 in the small intestine and liver (Figure 4). This might be because the basal level of CYP3A was already at such a high level that further increase in mRNA would not lead to a significant change of protein level.

### Effect of *Sophora* Extract on P-gp Expression in the Intestine and Liver

Effects of *Sophora* treatment on the intestinal and hepatic P-gp, an ATP-binding cassette (ABC) transporter also known as multidrug resistance protein (MDR) 1, expressions were examined. Two specific primers for the mdr1 gene subunits, mdr1a and mdr1b, were used for the measurement of P-gp mRNA levels. Figure 3(C) shows that, when compared to the control, *Sophora* treatment dose-dependently increased the intestinal mRNA level of mdr1a, with 80% (*P* = 0.073) and 125% (*P* = 0.003) enhancement in the low- and high-dose groups, respectively. There was no significant difference in the mRNA levels of mdr1b between the vehicle-treated and *Sophora*-treated rats (*P*>0.05, Figure 3(D)). In the liver, the mRNA expression of mdr1a was elevated in the *Sophora*-treated rats, with 38% (*P* = 0.098) and 75% (*P* = 0.017) increase in the low- and high-dose groups, respectively (Figure 3(C)). Meanwhile, as shown in Figure 3(D), high-dose *Sophora* treatment led to an increase in the hepatic mRNA level of mdr1b by 4.4-fold compared to the control (*P* = 0.002), while low-dose treatment did not show significant effect (*P*>0.05).

**Figure 5:**
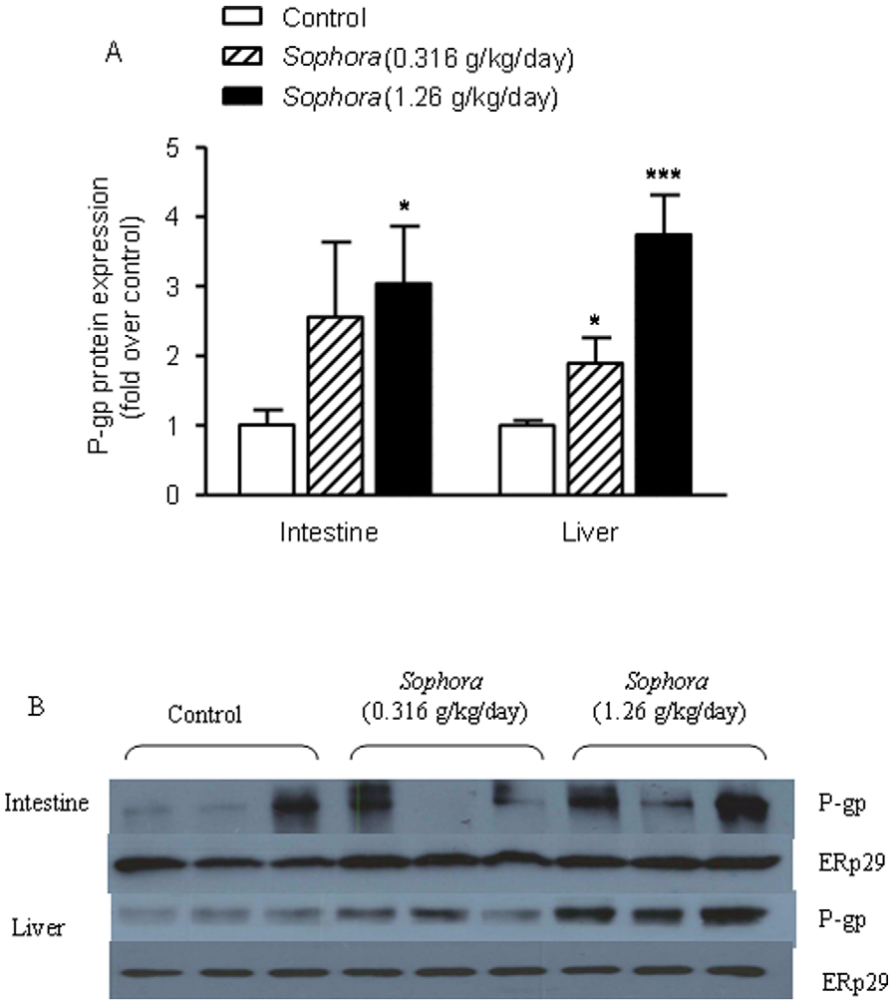
Effect of *Sophora* extract on the expression of intestinal and hepatic P-gp. (A) Statistical data analysis of Western blotting results, (B) image of Western blotting results. P-gp levels were measured using the anti-rat C219 antibody as described under *Materials and Methods*. ERp29 was used as control for the normalization of P-gp density. Bars represent mean ± S.E.M. of fold relative to the values in control group (n = 7–8). Statistical significance is indicated as * *P*<0.05, *** *P*<0.001, compared to control.

Figure 5(B) shows that P-gp was detected in both intestinal BBM and liver microsomes. Both intestinal and hepatic P-gp protein levels were elevated in the *Sophora*-treated rats. Compared to the control, low-dose *Sophora* treatment increased the intestinal and hepatic P-gp levels by 150% (*P* = 0.183) and 89% (*P* = 0.032), respectively (Figure 5(A)). The effects of high-dose treatment were even more significant, showing 2-fold (*P* = 0.034) and 2.7-fold (*P*<0.001) enhancements in the intestine and liver respectively (Figure 5(A)).

### Effect of *Sophora* Extract on Hepatic CYP3A Activity

**Figure 6:**
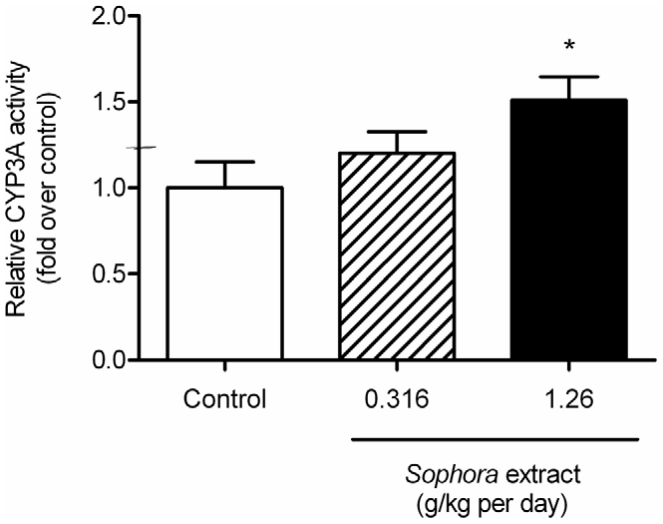
Effect of *Sophora* extract on the CYP3A activity in liver. The CYP3A activity was measured using a luminescent assay (P450-Glo). Bars represent mean ± S.E.M. of fold relative to the values in the control group (n = 9). Statistical significance is indicated as * *P*<0.05, compared to control.

The hepatic CYP3A activity was measured using a luminescent assay. Figure 6 reveals that the CYP3A activity in the liver was up-regulated after treatment with *Sophora* extract, showing 20% (*P* = 0.315) and 51% (*P* = 0.024) enhancements in the low- and high-dose groups, respectively.

## Discussion

Clinical and experimental evidence of herb-drug interactions have suggested that the combined use of herbs and prescribed drugs can potentially attenuate drug efficacy and/or enhance toxicity [29]. This is particularly important for patients suffering from chronic infections such as HIV/AIDS patients who are dependent on life-long antiretroviral treatment. It has been demonstrated that prolonged co-administration of health products, such as milk thistle, St John's wort and vitamin C supplements, will lead to a decrease of plasma indinavir concentrations with AUC reduction up to 57% [15], [30], [31]. For safety reason, it is thus important to evaluate the potential pharmacokinetic interaction between natural products and oral pharmaceutics in animals before conducting studies in human. In this study, the interaction between a herb, namely, *S. flavescens* and a commonly used antitretroviral, as exemplified by indinavir, a PI, was investigated in rats. Our findings demonstrated that co-administration of *S. flavescens* extract and indinavir (7-day) significantly decreased the plasma concentrations of the latter. In the *Sophora*-treated rats, the amount of faecal indinavir increased, whereas the urinary excretion markedly decreased. Biochemical studies suggested that the interaction was associated with the induction of intestinal and hepatic P-gp and CYP3A. To the best of our knowledge, this is the first report to demonstrate the impact of *S. flavascens* on the multiple-dose pharmacokinetics of indinavir.

When the *Sophora* extract was fractionated by partitioning with organic solvents, an ethyl acetate-soluble (EA) fraction was obtained. Treatment of rats with this *Sophora* EA fraction had no effect on indinavir pharmacokinetics, suggesting that the constituents of the EA fraction (mainly prenylated flavonoids) were unlikely to have contributed to the pharmacokinetic interaction. It is speculated that the herb-drug interaction may be mediated by the alkaloidal components of the plant. Such a speculation was supported by the observation that co-administration with oxymatrine capsule significantly decreased the plasma indinavir concentrations, with 61% reduction in AUC_0-∞_. Although other active ingredients may be present in the extract, oxymatrine is definitely an important component of *S. flavascens* associated with the pharmacokinetic interaction with indinavir. Since the experimental dose of oxymatrine is equivalent to a human dose of 0.9 g/day, the use of Marine capsule in HIV patients receiving indinavir should be cautioned.

Metabolically indinavir is designated as a Class 2 drug (low solubility/high permeability/extensive metabolism) [32]. The compound is highly permeable across the epithelium, as suggested by a rapid absorption rate (*T*_max_: 30–35 min) after oral administration [12]. The *T*_max_ value of indinavir determined in our study, ranging from 30 to 37 min, was consistent with values published in the literature. On the other hand, the bioavailability of indinavir is largely limited by efflux through intestinal P-gp and first-pass metabolism by CYP3A [14], [33], with the reported oral bioavailability in rats of 26%. In the present study, plasma indinavir concentrations decreased after co-administration of *Sophora* extract, resulting in a reduction of C_max_ and AUC_0-∞_. The decrease of C_max_ suggested that *S. flavescens* could enhance intestinal drug efflux and/or first-pass metabolism, thereby reducing the portion of indinavir entering systemic circulation. A similar observation was reported in the study between St. John's wort and indinavir [16], in which both intestinal and hepatic first-pass metabolism contributed to the reduction of indinavir bioavailability. Our findings also showed a dose-dependent increase in the CL/F value of indinavir after *Sophora* treatment, which can be interpreted as an enhancement of systemic clearance, reduction of absorption, and/or induction of first-pass metabolism. The contribution of each of these components to the decrease of indinavir exposure by *S. flavescens* remains to be characterized.

In our study urinary indinavir contents dropped in *Sophora*-treated rats (24-h), when compared to the control. Since urinary excretion of a drug correlates closely with its plasma concentration, this observation can reasonably be explained by the remarkable reduction of plasma indinavir (AUC) after *Sophora* treatment. On the other hand, the faecal indinavir content in *Sophora*-treated rats was higher than that of the control. The elevation was likely caused by the enhanced efflux of indinavir through an induction of intestinal P-gp (see below). In addition, since biliary excretion is also involved in the excretion of indinavir [12], the role of biliary pathway in the pharmacokinetic interaction remains to be investigated.

The CYP metabolic enzyme system and drug transporters are known to be inducible and they are often involved in herb-drug interactions [34]. Previous studies have established the pivotal roles of P-gp and CYP3A in the absorption and metabolism of indinavir [13], [14]. Plasma indinavir exposure decreased after pre-treatment with dexamethasone in rats, and the effect was attributed to the intestinal and hepatic first-pass metabolism through an induction of CYP3A and P-gp [33]. Our findings showed that *Sophora* treatment could induce the mRNA expression of CYP3A1 in the small intestine and liver, but had little effect on CYP3A2. Similar results were reported using dexamethasone as the ligand of pregnane X receptor (PXR) [35], which is a ligand-activated transcriptional regulator for drug metabolism and plays a dominant role in regulating CYP3A transcription [36]. Cell-based experiments have suggested that *S. flavescens* could induce CYP3A4 mRNA expression via the activation of PXR, and *N*-methylcytisine was identified as an active principle [10]. Thus, our observation of elevated CYP3A1 mRNA levels by *S. flavescens* might also be caused by PXR activation. Although protein expressions of CYP3A1 and CYP3A2 in the intestine and liver were not affected by *Sophora* treatment, hepatic CYP3A activity appeared to be elevated after *Sophora* treatment, which was consistent with the reported inductive effect of *S. flavescens* on CYP3A [7], [8]. The up-regulation of CYP3A activity enhanced indinavir metabolism and hence facilitated drug elimination, which can partially explain the decreased concentrations of indinavir.

Another determining factor involved in the herb-drug interaction is the P-gp. Indinavir is a known substrate of P-gp [14]. After co-administration of *Sophora* extract, dose-dependent increases in the intestinal and hepatic mRNA levels of P-gp were observed. Although the regulation of P-gp was complex and variable among tissues [35], the concurrent induction of CYP3A and P-gp in the small intestine and liver has previously been reported [33], [37]. Recent molecular investigations have discovered that P-gp (MDR1) transcription is regulated by two nuclear receptors, the PXR and the constitutive androstane receptor (CAR) [38], [39], which are also transcription regulators for CYP3A. It is thus speculated that the chemical ingredients in *S. flavescens* that can induce CYP3A expression may also induce P-gp via the activation of common transcription factors. The alteration of P-gp protein levels after *Sophora* treatment was in consensus with the elevation of mRNA contents. Since intestinal P-gp mediates drug efflux and limits the bioavailability of its substrates, the induction of intestinal P-gp by *S. flavescens* was likely contributory to the decrease of plasma indinavir, as well as the increase of faecal indinavir. In addition, interplay between P-gp and CYP3A have been demonstrated [40], [41]. By extruding the drug from the enterocytes to intestinal lumen during absorption, intestinal P-gp can increase the residence time for exposure to intestinal CYP3A, and consequently enhance its metabolism. A previous study showed that during the transport of indinavir in Caco-2 cells expressing CYP3A4, P-gp efflux resulted in more metabolites produced from indinavir [14]. Likewise, in our study, the increased level of intestinal P-gp, acting with CYP3A, may further enhance the intestinal first-pass metabolism of indinavir and lead to the dramatic decrease of its plasma concentrations. On the other hand, unlike intestinal P-gp, hepatic P-gp unlikely had significant effect on hepatic metabolism of indinavir [33]. It is evident that P-gp and CYP3A cannot fully account for the entire process of indinavir absorption and metabolism, and there must be other mediators that remain to be identified.

In the guidelines recommended by the Food and Drug Administration (FDA) for industry to study drug-drug interaction, *in vitro* CYP-based metabolism information should be obtained in the early stage [42]. In fact, most pharmaceutical companies use mass screening for potential CYP inhibitors by applying enzyme inhibition kinetics studies (calculating IC_50_, Km, Vmax, and Ki etc.) to illustrate the inhibition potential [43]. Our previous study using human pooled liver microsome and specific CYP3A4 isoform showed that *Sophora* extract inhibited CYP3A4 activity, and the EA fraction was the active fraction (IC_50_ of 5.0 µg/mL), implying that the *Sophora* EA fraction might exert influence on the pharmacokinetics of CYP3A substrate, such as indinavir. However, further experiments showed that the interaction between EA fraction (at highest dose of 0.328 g/kg) and indinavir was not observed *in vivo* (data not shown). It's thus concluded that the *in vitro* CYP enzyme inhibitory effects of herbal extracts may not necessarily carry significant impacts on the drugs' pharmacokinetics *in vivo*. In studying the induction potentials, *ex vivo* approach has been commonly used by measuring the CYP enzymes' expression and activity (probe substrate method) in liver after long-term (7 days or 14 days) administration of the herbal extract. A number of herbal medicines have been demonstrated to show inductive effects, including *S. flavescens* [7], [44]. It is however still uncertain if *ex vivo* effect could be directly extrapolated to *in vivo* conditions. Furthermore, *ex vivo* method tends to neglect the potential effects on the absorption of oral pharmaceuticals, such as inhibition or induction of intestinal transporters. When the drug is a dual substrate for CYP enzyme and transporter, such as indinavir (CYP3A and P-gp substrates), *ex vivo* study might not fully characterize the potential of herb-drug interactions involving the interplay of CYP enzyme(s) and transporter(s). It is against these backgrounds that the present pharmacokinetic study was conceptualized. Nevertheless, the underlying mechanisms of the interaction revealed in this study are far from fully understood. The potential role of gene regulation and the interplay between CYP3A and P-gp are yet to be uncovered, and an *in vitro* cell-based study can be useful for delineating underlying mechanism. Moreover, studies using human subjects or human-derived specimens remain to be conducted to illustrate the clinical significance as species difference may be present.

In summary, an animal model for characterizing plasma pharmacokinetics, excretion, and interaction mechanisms in herb-drug interactions is presented. Concomitant administration of *S. flavescens* or oxymatrine resulted in a remarkable reduction in plasma indinavir concentrations in rats, while the *Sophora* ethyl acetate fraction had no effect. The pharmacokinetic impact on indinavir was, at least in part, attributed to the induction of CYP3A and P-gp in the small intestine and liver, as well as the up-regulation of CYP3A activity. In spite of the presence of interspecies difference, the pharmacokinetic interaction demonstrated in the animal model implies that significant clinical consequence might occur during concomitant administration of *S. flavescens* and indinavir, especially in HIV/AIDS patients in whom antiviral resistance might rapidly develop under sub-optimal plasma indinavir concentrations. Therefore, patients receiving indinavir might be cautioned against the intake of *S. flavescens* extract or *Sophora*-derived products.

## Supporting Information

Figure S1 **HPLC-MS extracted ion chromatogram of indinavir (** ***m/z*** **614) and midazolam (I.S.,** ***m/z*** **326) in rat plasma obtained at 2 h after oral administration of indinavir (40 mg/kg).** The mass spectra of indinavir and midazolam are shown.
(TIF)

Figure S2 **Chromatograms of** ***Sophora*** **extract and fractions.** LC-MS analysis indicates the presence of flavonoids (area B) in the ethyl acetate fraction, whereas the water fraction mainly contains alkaloids (area A); the butanol fraction contains both flavonoids and alkaloids. Oxymatrine was identified by comparison with a standard. Other ingredients were tentatively identified by their LC-MS/MS characters.
(TIF)

## References

[1] National Committee of Chinese Pharmacopoeia (2010). Pharmacopoeia of the People's Republic of China.

[2] Chen X, Yi C, Yang X, Wang X (2004). Liquid chromatography of active principles in *Sophora flavescens* root.. J Chromatogr B Analyt Technol Biomed Life Sci.

[3] Zhang L, Xu L, Xiao SS, Liao QF, Li Q (2007). Characterization of flavonoids in the extract of *Sophora flavescens* Ait. by high-performance liquid chromatography coupled with diode-array detector and electrospray ionization mass spectrometry.. J Pharm Biomed Anal.

[4] Sun M, Han J, Duan J, Cui Y, Wang T (2007). Novel antitumor activities of Kushen flavonoids in vitro and in vivo.. Phytother Res.

[5] Sun H, Li L, Shang L, Zhao D, Dong D (2008). Cardioprotective effects and underlying mechanisms of oxymatrine against Ischemic myocardial injuries of rats.. Phytother Res.

[6] Liu Y, Zhang XJ, Yang CH, Fan HG (2009). Oxymatrine protects rat brains against permanent focal ischemia and downregulates NF-kappaB expression.. Brain Res.

[7] Ueng YF, Chen CC, Tsai CC, Soucek P (2009). Differential inductive profiles of hepatic cytochrome P450s by the extracts of *Sophora flavescens* in male and female C57BL/6JNarl mice.. J Ethnopharmacol.

[8] Ueng YF, Tsai CC, Lo WS, Yun CH (2010). Induction of hepatic cytochrome P450s by the herbal medicine *Sophora flavescens* extract in rats: impact on the elimination of theophylline.. Drug Metab Pharmacokinet.

[9] Yuan F, Chen J, Wu WJ, Chen SZ, Wang XD (2010). Effects of Matrine and Oxymatrine on Catalytic Activity of Cytochrome P450s in Rats.. Basic Clin Pharmacol Toxicol.

[10] Wang L, Li F, Lu J, Li G, Li D (2010). The Chinese herbal medicine *Sophora* flavescens activates pregnane X receptor.. Drug Metab Dispos.

[11] Chiba M, Hensleigh M, Nishime JA, Balani SK, Lin JH (1996). Role of cytochrome P450 3A4 in human metabolism of MK-639, a potent human immunodeficiency virus protease inhibitor.. Drug Metab Dispos.

[12] Lin JH, Chiba M, Balani SK, Chen IW, Kwei GY (1996). Species differences in the pharmacokinetics and metabolism of indinavir, a potent human immunodeficiency virus protease inhibitor.. Drug Metab Dispos.

[13] Chiba M, Hensleigh M, Lin JH (1997). Hepatic and intestinal metabolism of indinavir, an HIV protease inhibitor, in rat and human microsomes. Major role of CYP3A.. Biochem Pharmacol.

[14] Hochman JH, Chiba M, Nishime J, Yamazaki M, Lin JH (2000). Influence of P-glycoprotein on the transport and metabolism of indinavir in Caco-2 cells expressing cytochrome P-450 3A4.. J Pharmacol Exp Ther.

[15] Piscitelli SC, Burstein AH, Chaitt D, Alfaro RM, Falloon J (2000). Indinavir concentrations and St John's wort.. Lancet.

[16] Ho YF, Huang DK, Hsueh WC, Lai MY, Yu HY (2009). Effects of St. John's wort extract on indinavir pharmacokinetics in rats: differentiation of intestinal and hepatic impacts.. Life Sci.

[17] Sadler BM, Gillotin C, Lou Y, Stein DS (2001). Pharmacokinetic and pharmacodynamic study of the human immunodeficiency virus protease inhibitor amprenavir after multiple oral dosing.. Antimicrob Agents Chemother.

[18] Clevenbergh P, Durant J, Chaillou S, Dellamonica P (1999). HIV drug resistance and insufficient drug plasma levels as factors determining antiretroviral treatment failure.. AIDS Rev.

[19] Solas C, Basso S, Poizot-Martin I, Ravaux I, Gallais H (2002). High indinavir C_min_ is associated with higher toxicity in patients on indinavir-ritonavir 800/100 mg twice-daily regimen.. J Acquir Immune Defic Syndr.

[20] Lo Re V, Frank I, Gross R, Dockter J, Linnen JM (2007). Prevalence, risk factors, and outcomes for occult hepatitis B virus infection among HIV-infected patients.. J Acquir Immune Defic Syndr.

[21] He N, Chen L, Lin HJ, Zhang M, Wei J (2011). Multiple viral coinfections among HIV/AIDS patients in China.. Biosci Trends.

[22] Li CQ, Zhu YT, Zhang FX, Fu LC, Li XH (2005). Anti-HBV effect of liposome-encapsulated matrine in vitro and in vivo.. World J Gastroenterol.

[23] Azzam HS, Goertz C, Fritts M, Jonas WB (2007). Natural products and chronic hepatitis C virus.. Liver Int.

[24] Gui FS, Hua JH (2007). Clinical observations (76 cases) of eczema treated with Kushen Tablet.. Linchuang He Shiyan Yixue Zazhi (Chinese).

[25] Livak KJ, Schmittgen TD (2001). Analysis of Relative Gene Expression Data Using Real-Time Quantitative PCR and the 2^−ΔΔCT^ Method.. Methods.

[26] Kessler M, Acuto O, Storelli C, Murer H, Muller M (1978). A modified procedure for the rapid preparation of efficiently transporting vesicles from small intestinal brush border membranes. Their use in investigating some properties of D-glucose and choline transport systems.. Biochim Biophys Acta.

[27] Hamidi M (2006). Role of P-glycoprotein in tissue uptake of indinavir in rat.. Life Sci.

[28] Rezk NL, Crutchley RD, Yeh RF, Kashuba AD (2006). Full validation of an analytical method for the HIV-protease inhibitor atazanavir in combination with 8 other antiretroviral agents and its applicability to therapeutic drug monitoring.. Ther Drug Monit.

[29] Kennedy DA, Seely D (2010). Clinically based evidence of drug-herb interactions: a systematic review.. Expert Opin Drug Saf.

[30] DiCenzo R, Shelton M, Jordan K, Koval C, Forrest A (2003). Coadministration of milk thistle and indinavir in healthy subjects.. Pharmacotherapy.

[31] Slain D, Amsden JR, Khakoo RA, Fisher MA, Lalka D (2005). Effect of high-dose vitamin C on the steady-state pharmacokinetics of the protease inhibitor indinavir in healthy volunteers.. Pharmacotherapy.

[32] Wu CY, Benet LZ (2005). Predicting drug disposition via application of BCS: transport/absorption/elimination interplay and development of a biopharmaceutics drug disposition classification system.. Pharm Res.

[33] Lin JH, Chiba M, Chen IW, Nishime JA, deLuna FA (1999). Effect of dexamethasone on the intestinal first-pass metabolism of indinavir in rats: evidence of cytochrome P-450 3A and p-glycoprotein induction.. Drug Metab Dispos.

[34] Pal D, Mitra AK (2006). MDR- and CYP3A4-mediated drug-herbal interactions.. Life Sci.

[35] Mei Q, Richards K, Strong-Basalyga K, Fauty SE, Taylor A (2004). Using real-time quantitative TaqMan RT-PCR to evaluate the role of dexamethasone in gene regulation of rat P-glycoproteins mdr1a/1b and cytochrome P450 3A1/2.. J Pharm Sci.

[36] Goodwin B, Redinbo MR, Kliewer SA (2002). Regulation of cyp3a gene transcription by the pregnane X receptor.. Annu Rev Pharmacol Toxicol.

[37] Huang L, Wring SA, Woolley JL, Brouwer KR, Serabjit-Singh C (2001). Induction of P-glycoprotein and cytochrome P450 3A by HIV protease inhibitors.. Drug Metab Dispos.

[38] Geick A, Eichelbaum M, Burk O (2001). Nuclear receptor response elements mediate induction of intestinal MDR1 by rifampin.. J Biol Chem.

[39] Burk O, Arnold KA, Nussler AK, Schaeffeler E, Efimova E (2005). Antimalarial artemisinin drugs induce cytochrome P450 and MDR1 expression by activation of xenosensors pregnane X receptor and constitutive androstane receptor.. Mol Pharmacol.

[40] Benet LZ, Cummins CL, Wu CY (2004). Unmasking the dynamic interplay between efflux transporters and metabolic enzymes.. Int J Pharm.

[41] Kivistö KT, Niemi M, Fromm MF (2004). Functional interaction of intestinal CYP3A4 and P-glycoprotein.. Fundam Clin Pharmacol.

[42] CDER/CBER guidance for industry on drug interaction studies – study design, data analysis, and implications for dosing and labeling.. http://www.fda.gov/Drugs/GuidanceComplianceRegulatoryInformation/Guidances/ucm064982.htm.

[43] Yuan R, Madani S, Wei X, Reynolds K, Huang S-M (2002). Evaluation of P450 probe substrates commonly used by the pharmaceutical industry to study *in vitro* drug interactions.. Drug Metab Dispos.

[44] Agus HH, Tekin P, Bayav M, Semiz A, Sen A (2009). Drug interaction potential of the seed extract of Urtica urens L. (dwarf nettle).. Phytother Res.

