# Supplementary figures and images for: Impact of the Herbal Medicine Sophora flavescens on the Oral Pharmacokinetics of Indinavir in Rats: The Involvement of CYP3A and P-Glycoprotein
Source: PLoS One. 2012 Feb 16;7(2):e31312. doi: 10.1371/journal.pone.0031312 (PMC3281083; doi:10.1371/journal.pone.0031312)

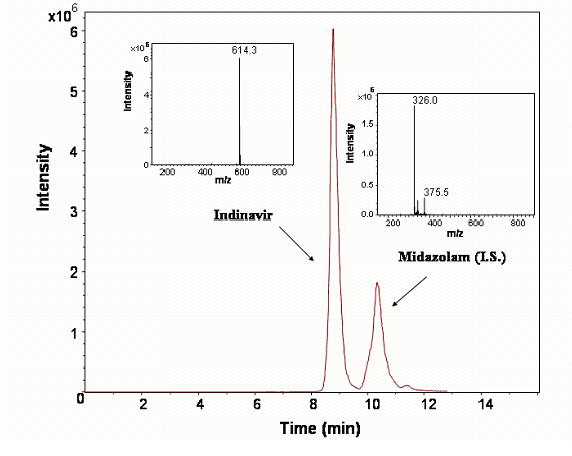

Supplement: Figure S1 — HPLC-MS extracted ion chromatogram of indinavir ( m/z 614) and midazolam (I.S., m/z 326) in rat plasma obtained at 2 h after oral administration of indinavir (40 mg/kg). The mass spectra of indinavir and midazolam are shown. (TIF) [file pone.0031312.s001.tif]

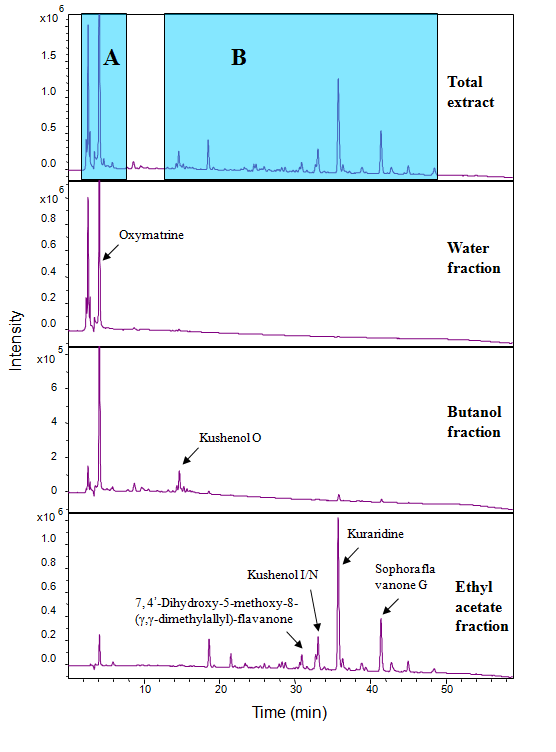

Supplement: Figure S2 — Chromatograms of Sophora extract and fractions. LC-MS analysis indicates the presence of flavonoids (area B) in the ethyl acetate fraction, whereas the water fraction mainly contains alkaloids (area A); the butanol fraction contains both flavonoids and alkaloids. Oxymatrine was identified by comparison with a standard. Other ingredients were tentatively identified by their LC-MS/MS characters. (TIF) [file pone.0031312.s002.tif]
